# Ocular Blood Flow Changes Impact Visual Acuity Gain after Surgical Treatment for Idiopathic Epiretinal Membrane

**DOI:** 10.3390/jcm9061768

**Published:** 2020-06-07

**Authors:** Felix Rommel, Max P. Brinkmann, Jan A. M. Sochurek, Michelle Prasuhn, Salvatore Grisanti, Mahdy Ranjbar

**Affiliations:** 1 Department of Ophthalmology, Ratzeburger Allee 160, 23538 Lübeck, Germany; michelle.prasuhn@uksh.de (M.P.); salvatore.grisanti@uksh.de (S.G.); eye.research101@gmail.com (M.R.); 2 Laboratory for Angiogenesis & Ocular Cell Transplantation, Ratzeburger Allee 160, 23538 Lübeck, Germany; max.brinkmann@gmx.de (M.P.B.); jan.sochurek@mailbox.org (J.A.M.S.)

**Keywords:** OCTA, idiopathic epiretinal membrane, choroidal perfusion, choriocapillaris, Sattler’s layer, Haller’s layer, retinal perfusion, vitreoretinal surgery

## Abstract

Background: Idiopathic epiretinal membrane (iERM) is a common eye disease, which can be treated by surgical removal of the fibrotic tissue. Morphological outcome is generally evaluated by optical coherence tomography (OCT). Here, we investigate the impact of surgery on hemodynamics of the posterior pole, using OCT angiography (OCTA). Methods: Patients with unilateral iERM and indication for treatment were included. OCT and OCTA images of the posterior pole were obtained preoperatively and 3-months after 23G vitrectomy with membrane peeling. Parameters of interest included full retinal perfusion, choriocapillaris perfusion (CCP), Sattler’s layer perfusion (SLP), and Haller’s layer perfusion, which were evaluated longitudinally and also compared to unaffected fellow eyes. Using these parameters, multiple regression analyses were used to predict visual outcomes. Results: Sixty-three iERM eyes were recruited, which initially showed a significant bigger central retinal thickness (*p* < 0.001) and total macular volume (TMV) (*p* < 0.001) compared to fellow eyes, while perfusion parameters were alike. Three months later, treated eyes had a statistically significant thicker subfoveal choroid (*p* = 0.006) and showed an increase of CCP (*p* = 0.003), while SLP decreased (*p* = 0.014). Lower preoperative TMV and higher SLP were associated with better postoperative visual outcome. Conclusion: In this OCTA study, iERM itself does not affect the choroidal circulation. However, preoperative choroidal circulation will be a biomarker to know the influence on the choroidal circulation after ERM surgery and may be considered as a predictor for visual outcome.

## 1. Introduction

Idiopathic epiretinal membrane (iERM) is a common macular disease in elderly people, characterized by fibrocellular tissue proliferation along the surface of the internal limiting membrane (ILM) [1]. The structural changes of the macular architecture lead to variable loss of visual acuity and metamorphopsia by causing vertical traction with macular thickening as well as tangential forces dragging the retina from its original position and displacing the vessels [2]. Pars plana vitrectomy with epiretinal membrane peeling is the standard of care to release the tension and restore the normal structure of the macula, commonly leading to visual acuity improvement and reduction of metamorphopsia [3]. Since studies suggest that ERM recurrence arises from remnant ERM components using the ILM as a scaffold, additional staining and ILM peeling is carried out by most surgeons [4]. However, in some cases, improvement of visual acuity is lacking, although ERM removal was successful; thus, an overall prognosis prediction model for postoperative best-corrected visual acuity (BCVA) is still needed [5].

With the recent development of optical coherence tomography angiography (OCTA), the retinal and choroidal vascular network can be assessed in vivo and in real time by creating slab-segmented angiograms, without the need for dye injection [6,7,8]. Only a few studies have evaluated vascular changes due to ERM formation and ERM removal by using OCTA, focusing especially on retinal microvascular impairment [9,10,11,12,13]. However, the role of the choroid in ERM pathogenesis is not well-known, although recent OCT- and OCTA-based studies suggest choroidal alterations from ERM and following ERM-ILM peeling [14,15,16].

The present study aimed to investigate structural and functional vascular alterations of the retina and the choroidal sublayers in patients with ERM. Furthermore, changes of OCT and OCTA parameters following pars plana vitrectomy with ERM-ILM peeling were analyzed and evaluated as predictive prognostic markers for the postoperative visual outcome.

## 2. Methods

### 2.1. Study Participants

The participants for this prospective observational study were enrolled at the Department of Ophthalmology of the University of Lübeck. The study was approved by the institutional review board (vote reference number 18-255) and adhered to the tenets of the Declaration of Helsinki. All participants received detailed information about the study and written informed consent was obtained individually by each participant before enrolment. Only subjects with unilateral, idiopathic, and most importantly, symptomatic ERM and presence of metamorphopsia, which were scheduled for vitrectomy with ERM and ILM peeling on the next day, were included. Exclusion criteria were: (1) ERM secondary to other pathologies such as retinal detachment, laser photocoagulation, cryotherapy, diabetic retinopathy, uveitis, trauma or vascular occlusion; (2) any history of previous ocular surgery, except for uncomplicated cataract surgery; (3) evidence or history of systemic disorders, including cardiovascular diseases, antihypertensive drug use, as well as diabetes mellitus; (4) history of ocular diseases in the healthy fellow eyes.

### 2.2. Study Protocol

At baseline, all participants underwent a thorough examination including refraction, best-corrected visual acuity (BCVA) in Snellen, intraocular pressure (IOP), axial length (AL) measurement, slit-lamp biomicroscopy, macular enhanced-depth imaging (EDI) OCT, as well as OCTA. The maximum permissible spherical and cylindrical aberration was ±3 and ±1 diopters respectively. Imaging was performed on all subjects using the Zeiss Cirrus HD-OCT (AngioPlex, CIRRUS HD-OCT model 5000, Carl Zeiss Meditec, Inc., Dublin, CA, USA) by a single, trained operator (F.R.). Each imaging session included EDI-OCT scans (10 × 10 mm^2^) and OCTA (3 × 3 mm^2^) volumetric scans of the posterior pole. Only scans with a signal strength ≥7, centered on the fovea, and without motion, as well as segmentation and projection artifacts, were considered to guarantee standardized analysis [6,17].

All iERM eyes underwent 23G pars plana vitrectomy with ERM and ILM peeling after staining with MembraneBlue-Dual^®^ (DORC Int., Zuidland, The Netherlands) under general anesthesia. The procedures were performed by a single, experienced vitreoretinal surgeon (M.R.). The periphery was checked with scleral indentation and a fluid-air exchange was performed at the end of the surgery. In cases of phakic eyes, vitrectomy was combined with phacoemulsification and lens implantation in the capsular bag. Three months after treatment, all participants underwent another thorough examination of both eyes including BCVA, slit-lamp biomicroscopy, macular EDI-OCT as well as OCTA. All OCT/OCTA imaging sessions were performed around noon to avoid the influence of physiological diurnal variations of perfusion metrics.

### 2.3. OCT and OCTA Data Analysis

Total macular volume (TMV) and central retinal thickness (CRT) were automatically acquired by the device, as defined by the Early Treatment Diabetic Retinopathy Study in the OCT scans [18]. SFCT was measured manually in EDI-OCT scans just below the fovea, extending perpendicularly from the hyperreflective Bruch’s membrane layer to the inner scleral border. Manual measurements were performed by two experienced graders (J.A.M.S. and M.P.) who were blinded to the clinical information of the examined eyes and results were averaged. On baseline examination, the iERM was graded (M.P.B.) according to a previous published ERM staging system and alterations of the central foveal bouquet (CB) were described as published by Govetto et al. [19,20].

**Figure 1:**
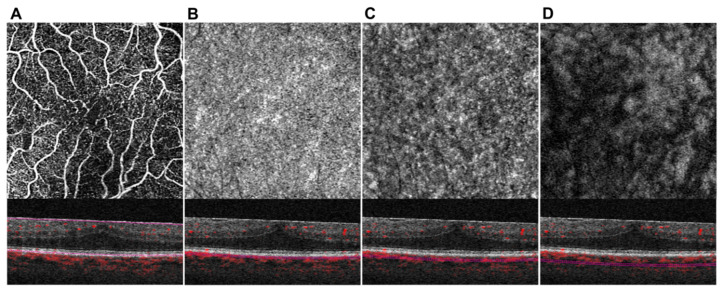
Optical coherence tomography angiography (OCTA)-Imaging of the posterior pole in a subject with idiopathic epiretinal membrane (iERM). En face angiogram and corresponding B-scan at the level of the full retina (**A**), choriocapillaris (**B**), Sattler’s layer (**C**), and Haller’s layer (**D**).

After acquisition, OCTA data were manually segmented (F.R.) in all B-Scans to get retinal and choroidal sublayer slabs. To avoid artifacts, the retinal vessels were analyzed as full retinal slabs (ILM to retinal pigment epithelium), since subjects with ERM are prone to segmentation errors within the retinal layers (Figure 1A) [17]. The choroidal sublayers were manually segmented to get 20 µm slabs of choriocapillaris (CC) (Figure 1B), Sattler’s layer (SL) (Figure 1C), and Haller’s layer (HL) (Figure 1D) according to previous published protocols [8,15,21,22]. All acquired en face images were exported into ImageJ (NIH, Version 1.48b, Bethesda, USA) for thresholding. Binarization was done by the Otsu method, which is an automatic threshold selection from grey-level histograms [23]. As suggested by Nicolò et al., CC perfusion (CCP) was calculated by scoring the percentage of white pixels, while for SL perfusion (SLP) and HL perfusion (HLP) the values of black pixels were taken into account [24].

### 2.4. Statistical Analysis

Statistical calculations were performed using IBM SPSS (Version 24.0, Chicago, IL, USA) and Prism GraphPad (Version 8.0, La Jolla, CA, USA). Snellen BCVA was converted to the logarithm of the minimum angle of resolution (logMAR). Quantitative variables were summarized as mean and standard deviation (SD) and qualitative variables as frequency and percentage. The Shapiro–Wilk test was used to check for normality of the obtained data. Baseline and follow-up values of the same eye were compared using a paired t-test. Differences between iERM eyes and healthy fellow eyes were assessed by an unpaired t-test. A multiple regression was carried out to investigate whether retinal and choroidal OCT/-A baseline parameter could significantly predict the visual outcome after surgery. For all tests, values of *p* < 0.05 were considered statistically significant.

## 3. Results

A total of 63 eyes with unilateral iERM and 63 healthy fellow eyes were included in the analysis. Table 1 reports clinical and demographic characteristics of the enrolled patients. Twenty-eight (44.4%) female and 35 (55.6%) male participants were included, with a mean age of 70.79 ± 7.86 years.

Anatomical and functional parameters of the iERM eyes and the fellow eyes at baseline examination are reported in Table 2. iERM eyes showed a statistically significantly worse BCVA and a higher TMV, as well as CRT, compared to the healthy fellow eyes. Perfusion values of the retina and the choroidal sublayers did not differ between both eyes.

Table 3 presents the anatomical and functional findings of the iERM eyes before and 3 months after treatment. The follow up examination revealed a significantly improved BCVA (0.46 ± 0.27 logMAR to 0.19 ± 0.19 logMAR; *p* < 0.001), while CRT (470.4 ± 81.02 µm to 405.2 ± 51.83 µm; *p* < 0.001) and TMV (10.43 ± 1.08 mm^3^ to 9.46 ± 0.61 mm^3^; *p* < 0.001) decreased. Furthermore, CCP rose from 37.86 ± 5.55% to 39.4 ± 5.77% (*p* = 0.003) and SLP dropped from 68.54 ± 8.92% to 67.76 ± 8.65% (*p* = 0.014) 3 months after surgery.

The untreated healthy fellow eyes did not show any statistically significant differences between baseline and follow up examination (Table 4). Comparison between affected eyes and healthy fellow eyes 3 months after treatment still showed significant differences in BCVA, CRT and TMV (Table 5). Subgroup analysis of phakic and pseudophakic patients revealed similar changes in terms of retinal and choroidal parameters due to combined phaco-vitrectomy or sole vitrectomy, respectively. The visual outcome 3 months after surgery in regard to BCVA improvement did not differ between both groups (phakic: −0.258 ± 0.183 logMAR, pseudophakic: −0.278 ± 0.193 logMAR, *p* = 0.96).

**Figure 2:**
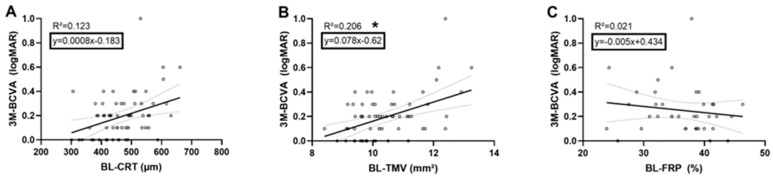
Linear regression model showing the 3-months (3M) best corrected visual acuity (BCVA) dependency of each central retinal thickness (CRT) (**A**), total macular volume (TMV) (**B**), and full retinal perfusion (FRP) (**C**) at baseline (BL). Black line represents the linear regression line; dotted line shows the upper and lower bound of the 95% confidence interval. * signifies *p* < 0.05.

**Figure 3:**
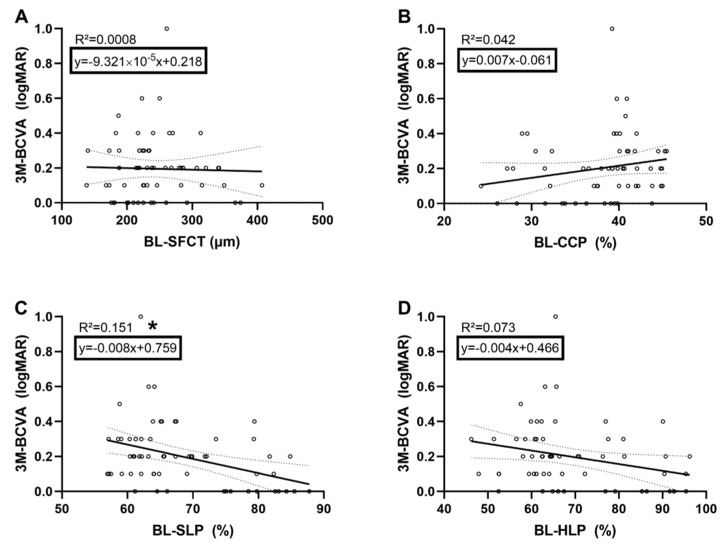
Linear regression model showing the 3-months (3M) BCVA dependency of each SFCT (**A**), CCP (**B**), SLP (**C**), and HLP (**D**) at baseline (BL). Black line represents the linear regression line; dotted line shows the upper and lower bound of the 95% confidence interval. * signifies *p* < 0.05.

Multiple regression analyses were carried out to investigate preoperative retinal and choroidal parameters as predictors of the postoperative BCVA in iERM eyes. The results of the regression analysis with retinal parameters indicated that the model explained 33.6% of the variance and that the model was a significant predictor of postoperative BCVA, F(3,33), *p* = 0.003. While TMV (B = 0.128, *p* = 0.004) contributed significantly to the model, CRT (B = 0.0003, *p* = 0.592) and FRP (B = −0.001, *p* = 0.860) did not (Figure 2). The regression analysis with choroidal baseline parameters indicated that the model explained 18.9% of the variance and that the model was a significant predictor of postoperative BCVA, F(4,55), *p* = 0.019. While SLP (B = −0.019, *p* = 0.006) contributed significantly to the model, CCP (B = −0.009, *p* = 0.214) and HLP (B = 0.005, *p* = 0.214) as well as SFCT (B = 0.001, *p* = 0.77) did not (Figure 3). Statistical significance of both regression models was independent of the lens status.

ERM eyes were grouped by the stage of the disease and baseline SLP values were compared across the four groups. Analysis of variance did not reveal any statistically significant difference between the groups. Another multiple regression analysis was carried out to investigate ERM stage and baseline SLP as predictors of the postoperative BCVA in the ERM eyes. The results of the regression indicated that the model explained 26.2% of the variance and that the model was a significant predictor of postoperative BCVA, F(2,58), *p* < 0.001. Both, ERM stage (B = 0.057, *p* = 0.004) and SLP (B = −0.007, *p* = 0.004) contributed significantly to the model.

## 4. Discussion

In this prospective OCTA-based study, we investigated retinal and choroidal vascular alterations in eyes with iERM as well as their reversibility after pars plana vitrectomy with ERM-ILM peeling. Furthermore, regression models, based on retinal and choroidal parameters, were carried out to identify predictive prognostic markers for the postoperative visual outcome. To the best of our knowledge, this is the first study demonstrating significant changes of SLP after iERM-surgery and the prognostic value of preoperative SLP as a predictor for the postoperative BCVA.

Idiopathic ERM is a frequent cause of reduced visual acuity with metamorphopsia and distinctly impairs patient quality of life [25]. ERM formation commonly exposes the macular to anteroposterior forces with macular thickening as well as tangential forces with vessel displacement [26,27]. Previous OCTA studies investigated alterations of the foveal avascular zone (FAZ) in eyes with ERM, demonstrating a smaller FAZ area in the superficial capillary plexus (SCP) and deep capillary plexus (DCP) [10,13,28]. They supposed that centripetal retinal displacement and the deformation of macular vascular integrity results in decreases of the FAZ area and parafoveal vessel density (VD) with a central vessel crowding. However, reported retinal perfusion values in eyes affected by ERM are quite different. Kim et al., as well as Chen et al., investigated the parafoveal VD in SCP and DCP, demonstrating reduced VD in both plexuses compared to unaffected fellow eyes [10,11]. Taking into account that mechanical stress due to ERM directly affects the inner retinal layers, it would be plausible that the SCP would be primary affected. However, Lin et al. found lower flow signals only in the DCP, while SCP only showed tortuous alterations [29]. Conversely, Nelis et al. demonstrated a significant increase of the foveal and parafoveal VD of iERM eyes in both retinal plexus. These overall controversial results in vessel quantification measurements could be due to false retinal layer segmentation. In ERM patients, the foveal architecture is distorted and slab segmentation is a serious issue, with segmentation errors occurring in 69.2% of ERM eyes [17,30]. Primarily the inner plexiform layer is prone to inaccurate segmentation; however, it is the most important layer for correct visualization and quantification of the SCP and DCP [6,7]. In order to avoid the potential bias of misalignment in the inner retina, we only investigated perfusion of the full-thickness retina slab in this study. ERM eyes showed a slightly lower mean FRP compared to unaffected fellow eyes at baseline examination, but without statistical significance (*p* = 0.383). Three months after surgery, overall retinal perfusion of the iERM group did not show any significant difference compared to preoperative values. Mastropasqua et al. evaluated SCP changes occurring after ERM-ILM peeling using OCTA [9]. Likewise, the SCP VD was stable throughout the follow-up examinations at 1 week and 1 month. However, analyzing individual sectors of SCP, they found a significant reduction of VD. In contrast to this study, Mastropasqua et al. evaluated automatically splatted 6 × 6 mm^2^ OCTA images; hence we may have missed out on those sectoral changes by analyzing the whole 3 × 3 mm^2^ en face image. Interestingly, another study group, who also investigated SCP perfusion changes after surgery, did not find significant perfusion changes after 1 month follow-up [12]. However, at 6 months follow-up, perfusion parameters significantly improved compared to baseline values. They suggested that forces exerted from ERM could lead to a temporary occlusion of small vessels and, after vitrectomy, the microvasculature needs a longer time to recover completely. We may have missed out on postoperative FRP changes by choosing a follow-up period of 3 months. However, previous studies demonstrated that the decisive retinal alterations occur within the first 3 months after surgery [31].

So far, inner-retinal layer alterations have gained attention in eyes with ERM, because ERM is primarily an inner retinal disease. However, with the introduction of EDI-OCT, as well as OCTA, choroidal involvement of ERM can be assessed more accurately. In this study, we analyzed SFCT and perfusion values of choroidal substructures at baseline and 3 months after ERM-ILM peeling. Compared to the unaffected fellow eyes, iERM eyes showed slightly reduced SFCT and CCP without reaching statistical significance. However, 3 months after surgery, the choroid thickened (*p* = 0.006), while CCP increased (*p* = 0.003) and SLP decreased (*p* = 0.014). The influence of ERM on choroidal thickness values has been described by Michalewska et al., who demonstrated a decrease of SFCT 3 months after vitrectomy with ERM removal and ILM peeling, contrary to our data [32]. Kang et al., who also investigated SFCT in patients after vitrectomy for ERM, did not find any significant difference in SFCT values after surgery [33]. In our study, the thickening of the SFCT could be related to the significant increase in CCP, since alterations in choroidal thickness reflect the status of the vasculature within [34]. Yu et al. support these findings as they reported a reduced CCP in ERM eyes compared to unaffected fellow eyes, which was reversible by surgery [14].

So far it is unclear, to what extent ERM-associated traction influences the microvasculature in SL and HL, since the vessel size increases from CC to HL. To the best of our knowledge, this is the first study reporting a significant decrease of SLP 3 months after surgery, while HLP stayed steady. An influence of ERM on deeper choroidal sublayers has been reported in terms of changes in the usual diurnal pattern of SLP an HLP [15]. ERM might lead to choroidal vascular involvement through a variety of possible mechanisms. The anteroposterior forces can affect the retinal pigment epithelium (RPE) as well as the choroid directly by focal dilation of choroidal vessel [16,35]. Through the release of mechanical stretching of the RPE after ERM-ILM removal, the local level of vascular endothelial growth factor (VEGF) could change, which might lead to alterations in choroidal permeability and vascular rearrangement [36]. The remodeling of the physiological conditions after surgery could possibly explain the increase of CCP and decrease of SLP as a shift of blood flow from the deeper choroid to the CC. Another factor that must be taken into account is the status of vitreous oxygenation after vitrectomy. The retina as well as the choroid is exposed to a higher oxygenated environment after removal of the oxygen-consuming vitreous, what may result in alteration of perfusion. It should be mentioned, that unaffected fellow eyes did not show any changes in terms of anatomical or functional parameters 3 months after baseline examination.

Furthermore, we carried out multiple regression models to investigate preoperative image parameters as predictors of the postoperative visual outcome in iERM eyes. TMV contributed significantly to the model and showed positive correlation with the postoperative BCVA (*p* = 0.004); thus a higher preoperative TMV led to worse visual outcome. The increase in TMV seen in eyes with ERM generally occurs from strong anteroposterior forces. Patients with lower TMV and less CRT are likely to suffer from an ERM with little traction. Therefore, these patients with low tractive ERM are unlikely to benefit from surgery.

In the regression analysis of preoperative choroidal parameters, SLP showed a statistically significant negative correlation with postoperative BCVA. In other words, higher preoperative SLP led to a better visual outcome 3 months after surgery. As we have suggested before, the release of mechanical tension from the ERM might lead to a remodeling of the physiological choroidal conditions with a perfusion shift from SL to CC. Therefore, the preoperative SLP could reflect the capacity of choroidal blood flow, which is able to shift into the smaller vessels of the CC. Interestingly, baseline SLP seems to be independent from the ERM stage. Therefore, it can be used as a universal biomarker. To the best of our knowledge, this is the first study reporting choroidal perfusion metrics as predictors of visual outcome after surgery for iERM.

The present study has some limitations, additional to those already discussed. The follow-up period after surgery was relatively short, since morphological changes could last up to 12 months after ERM peeling [31]. However, it has been reported that the decisive retinal alterations occur within the first 3 months after surgery. Furthermore, we included all stages of ERM, in order to avoid selection bias. However, severe retinal distortion due to thick and hyperreflective ERM could confound OCTA signals and measurements depending on the ERM stage. Finally, the manual record of SFCT may represent a potential bias. To reduce this possible confounding factor, all manual measurements were performed by two experienced graders in a masked fashion and the average values were used for statistical analysis.

## 5. Conclusions

In conclusion, we were able to demonstrate significant choroidal sublayer perfusion changes after surgery for iERM with an increase of CCP and a decrease of SLP, by using OCTA. Preoperative SLP could be a useful predictive marker for functional results, since a significant correlation with postoperative visual acuity was found.

## Figures and Tables

**Table 1:** Demographic and clinical characteristics of enrolled patients with iERM.

Parameter	iERM Eye	Fellow Eye
Gender (male/female), n (%)	35 (55.6%)/28 (44.4%)	
Age (years), mean ± SD	70.79 ± 7.86	
Eye (right/left), n (%)	34 (54%)/29 (46%)	29 (46%)/34 (54%)
Phakic/pseudophakic, n (%)	45 (71.4%)/18 (28.6%)	47 (74.6%)/16 (25.4%)
ERM stage, n (%)		
1	13 (20.6%)	-
2	14 (22.2%)	-
3	18 (28.6%)	-
4	18 (28.6%)	-
CB, n (%)		
0	41 (65.1%)	-
1	12 (19%)	-
2	6 (9.5%)	-
3	4 (6.4%)	-

iERM: idiopathic epiretinal membrane; SD: standard deviation; CB: central foveal bouquet.

**Table 2:** Anatomical and functional parameters of iERM and fellow eyes at baseline examination.

Parameter	iERM Eyes	Fellow Eyes	*p*-Value ^1^
AL (mm)	23.57 ± 1.02	23.67 ± 1.19	0.614
BCVA (logMAR)	0.46 ± 0.27	0.07 ± 0.08	<0.001
CRT (µm)	470.4 ± 81.02	322.2 ± 68.97	<0.001
TMV (mm^3^)	10.43 ± 1.08	8.78 ± 0.67	<0.001
SFCT (µm)	243 ± 56.9	255.6 ± 65.65	0.239
FRP (%)	36.15 ± 5.66	37.57 ± 7.77	0.383
CCP (%)	37.86 ± 5.55	39.47 ± 6.08	0.138
SLP (%)	68.54 ± 8.92	67.83 ± 8.45	0.659
HLP (%)	69.22 ± 13.11	69.33 ± 13.17	0.962

^1^ unpaired t-test; all data are presented as mean ± standard deviation; iERM: idiopathic epiretinal membrane; AL: axial length; BCVA: best corrected visual acuity; CRT: central retinal thickness; TMV: total macular volume; SFCT: subfoveal choroidal; FRP: full retinal perfusion; CCP: choriocapillaris perfusion; SLP: Sattler’s layer perfusion; HLP: Haller’s layer perfusion.

**Table 3:** Anatomical and functional parameters of iERM eyes before and 3 months after surgery.

Parameter	Baseline	3 months	*p*-Value ^1^
BCVA (logMAR)	0.46 ± 0.27	0.19 ± 0.19	<0.001
CRT (µm)	470.4 ± 81.02	405.2 ± 51.83	<0.001
TMV (mm^3^)	10.43 ± 1.08	9.46 ± 0.61	<0.001
SFCT (µm)	243 ± 56.9	259.8 ± 59.88	0.006
FRP (%)	36.15 ± 5.66	35.25 ± 7.11	0.249
CCP (%)	37.86 ± 5.55	39.4 ± 5.77	0.003
SLP (%)	68.54 ± 8.92	67.76 ± 8.65	0.014
HLP (%)	69.22 ± 13.11	69.39 ± 13.61	0.653

^1^ paired *t*-test; all data are presented as mean ± standard deviation; iERM: idiopathic epiretinal membrane; BCVA: best corrected visual acuity; CRT: central retinal thickness; TMV: total macular volume; SFCT: subfoveal choroidal; FRP: full retinal perfusion; CCP: choriocapillaris perfusion; SLP: Sattler’s layer perfusion; HLP: Haller’s layer perfusion.

**Table 4:** Anatomical and functional parameters of healthy fellow eyes at baseline and 3 months follow up examination.

Parameter	Baseline	3 Months	*p*-Value ^1^
BCVA (logMAR)	0.07 ± 0.08	0.08 ± 0.08	0.16
CRT (µm)	322.2 ± 68.97	321.4 ± 70.47	0.39
TMV (mm^3^)	8.78 ± 0.67	8.77 ± 0.68	0.165
SFCT (µm)	255.6 ± 65.65	253.4 ± 65.65	0.194
FRP (%)	37.57 ± 7.77	37.93 ± 7.25	0.89
CCP (%)	39.47 ± 6.08	39.34 ± 7.99	0.546
SLP (%)	67.83 ± 8.45	67.76 ± 8.45	0.569
HLP (%)	69.33 ± 13.17	69.52 ± 12.81	0.12

^1^ paired *t*-test; all data are presented as mean ± standard deviation; iERM: idiopathic epiretinal membrane; BCVA: best corrected visual acuity; CRT: central retinal thickness; TMV: total macular volume; SFCT: subfoveal choroidal; FRP: full retinal perfusion; CCP: choriocapillaris perfusion; SLP: Sattler’s layer perfusion; HLP: Haller’s layer perfusion.

**Table 5:** Anatomical and functional parameters of iERM and fellow eyes at 3 months follow up examination.

Parameter	iERM Eyes	Fellow Eyes	*p*-Value ^1^
BCVA (logMAR)	0.19 ± 0.19	0.08 ± 0.08	<0.001
CRT (µm)	405.2 ± 51.83	321.4 ± 70.47	<0.001
TMV (mm^3^)	9.46 ± 0.61	8.77 ± 0.68	<0.001
SFCT (µm)	259.8 ± 59.88	253.4 ± 65.65	0.569
FRP (%)	35.25 ± 7.11	37.93 ± 7.25	0.151
CCP (%)	39.4 ± 5.77	39.34 ± 7.99	0.97
SLP (%)	67.76 ± 8.65	67.76 ± 8.45	0.997
HLP (%)	69.39 ± 13.61	69.52 ± 12.81	0.96

^1^ unpaired *t*-test; all data are presented as mean ± standard deviation; iERM: idiopathic epiretinal membrane; BCVA: best corrected visual acuity; CRT: central retinal thickness; TMV: total macular volume; SFCT: subfoveal choroidal; FRP: full retinal perfusion; CCP: choriocapillaris perfusion; SLP: Sattler’s layer perfusion; HLP: Haller’s layer perfusion.
